# Nonlinearity-Guided Dual-Spectrum Ultrasonic Inversion for Attenuation-Independent Characterization of Subwavelength Coatings

**DOI:** 10.3390/s26113331

**Published:** 2026-05-24

**Authors:** Lei Wang, Cong Wan, Jiacheng Wang, Jianlin Xu, Yongfeng Song, Maodan Yuan

**Affiliations:** 1 Safety, Environment & Technology Supervision Research Institute, PetroChina Southwest Oil & Gasfield Company, Chengdu 610041, China; 2 State Key Laboratory of Precision Electronic Manufacturing Technology and Equipment, Guangdong University of Technology, Guangzhou 510006, China; 3 Sichuan Special Equipment Inspection Institute, Chengdu 610000, China

**Keywords:** ultrasonic testing, subwavelength coatings, layer-phase spectrum, nonlinearity-guided optimization, attenuation-independent inversion

## Abstract

The nondestructive characterization of coating thickness, acoustic velocity, and density is essential for industrial quality control. However, conventional ultrasonic reflection coefficient amplitude spectrum (URCAS)-based inversion methods typically require prior knowledge of acoustic attenuation, which is often unavailable for thin coatings and limits their practical applicability. To address this issue, a nonlinearity-guided dual-spectrum inversion framework is proposed by combining the URCAS with a layer-phase spectrum. It is found that the layer-phase spectrum exhibits strong nonlinear sensitivity to variations in acoustic velocity and density, which helps improve parameter identifiability. Based on this property, an improved particle swarm optimization algorithm is developed to enable simultaneous inversion of thickness, velocity, and density without explicit prior attenuation information. Finite-element simulations show that the conventional URCAS method yields mean relative errors exceeding 5%, whereas the proposed method reduces these errors to below 3% under the tested conditions. Experimental validation on eight industrial polytetrafluoroethylene (PTFE) coatings with thicknesses ranging from 20.89 μm to 120.11 μm (down to approximately 0.11 λ at 10 MHz) demonstrates that the proposed method achieves average relative errors within 10% and improves inversion accuracy by about 6% compared with amplitude-only approaches. The results indicate that the proposed attenuation-independent and nonlinearity-guided strategy provides an effective solution for the quantitative nondestructive evaluation of subwavelength coatings. The method is particularly suitable for thin coatings with unknown attenuation.

## 1. Introduction

Fluoropolymer coatings such as PTFE are widely used in electronic and industrial applications because of their waterproof, anti-corrosion, and wear-resistant properties. Their functional performance is strongly governed by thickness and density, making accurate nondestructive characterization essential for quality control [1]. At present, coating thickness and density are mainly evaluated using destructive methods. Thickness is typically measured via cross-sectional microscopy [2], while density is determined using the Archimedes method [3]. Although effective, these approaches inevitably damage samples and are unsuitable for in situ inspection or large-area industrial applications. Therefore, developing a nondestructive method for simultaneous characterization of coating thickness and density remains a critical challenge.

Time-domain ultrasonic methods based on the time-of-flight (TOF) principle are widely used for thickness measurement [4]. However, for subwavelength coatings, the strong overlap of interface echoes makes reliable separation extremely difficult, even with advanced signal processing techniques such as adaptive filtering and matching pursuit [5,6], which often involve high computational cost and remain sensitive to noise.

To overcome this limitation, frequency-domain methods have been developed based on the analysis of standing-wave resonance characteristics. Resonance frequency analysis has been applied to thin-walled structures [7], while filter-based interpretations and autocorrelation techniques have improved robustness under limited bandwidth and noise conditions [8,9]. In addition, phase-based approaches, such as the reflection-coefficient phase method, have demonstrated potential for characterizing ultrathin layer [10,11].

More importantly, most existing frequency-domain approaches fundamentally rely on identifiable resonance features in the spectrum. However, when the coating thickness falls within the subwavelength regime (i.e., thickness smaller than the acoustic wavelength), the resonance features become significantly weakened or even indistinguishable, making reliable parameter inversion challenging. In contrast, for relatively thick coatings, resonance peaks are more pronounced and acoustic attenuation can often be measured or reasonably estimated, allowing conventional URCAS-based methods to achieve satisfactory accuracy. This contrast highlights that current methods are less reliable for subwavelength coatings, where resonance information is insufficient and attenuation is difficult to obtain.

In ultrasonic spectral inversion, existing methods can be broadly categorized into amplitude-based and phase-based approaches. Amplitude-spectrum-based methods (e.g., transfer-function models [12], oblique-incidence analysis [13], and multilayer transmission-line models [14]) enable multiparameter inversion but generally require prior knowledge of acoustic attenuation, which is difficult to obtain in practical applications. Similarly, full-band inversion and PSO-based approaches [15,16,17,18,19,20] reduce reliance on resonance features but still depend on attenuation priors. In contrast, phase-spectrum-based methods, particularly those based on the layer-phase spectrum [21,22,23,24,25], are inherently less sensitive to acoustic attenuation and do not require prior attenuation information. However, they are typically limited to estimating thickness and acoustic velocity and cannot simultaneously determine density.

Therefore, a clear technical gap exists for subwavelength coatings: amplitude-based methods allow multiparameter inversion but depend on attenuation priors, whereas phase-based methods avoid attenuation dependence but cannot provide complete parameter estimation. To address this issue, this study proposes a nonlinearity-guided dual-spectrum inversion framework that integrates amplitude and phase information. By combining the complementary characteristics of the two spectra, the method reduces the reliance on attenuation while improving parameter identifiability. Specifically, the nonlinear dependence of the layer-phase spectrum on acoustic velocity and density is exploited to guide an improved particle swarm optimization algorithm, enabling simultaneous inversion of thickness, acoustic velocity, and density without requiring explicit attenuation priors.

The remainder of this paper is organized as follows. Section 2 presents the theoretical model and the proposed nonlinearity-guided inversion strategy. Section 3 validates the method through finite-element simulations. Section 4 reports experimental results on PTFE-coated samples. Section 5 concludes the paper.

## 2. Theoretical Basis and Inversion Methodology

### 2.1. Forward Model and the Limitations of Amplitude Spectrum

**Figure 1:**
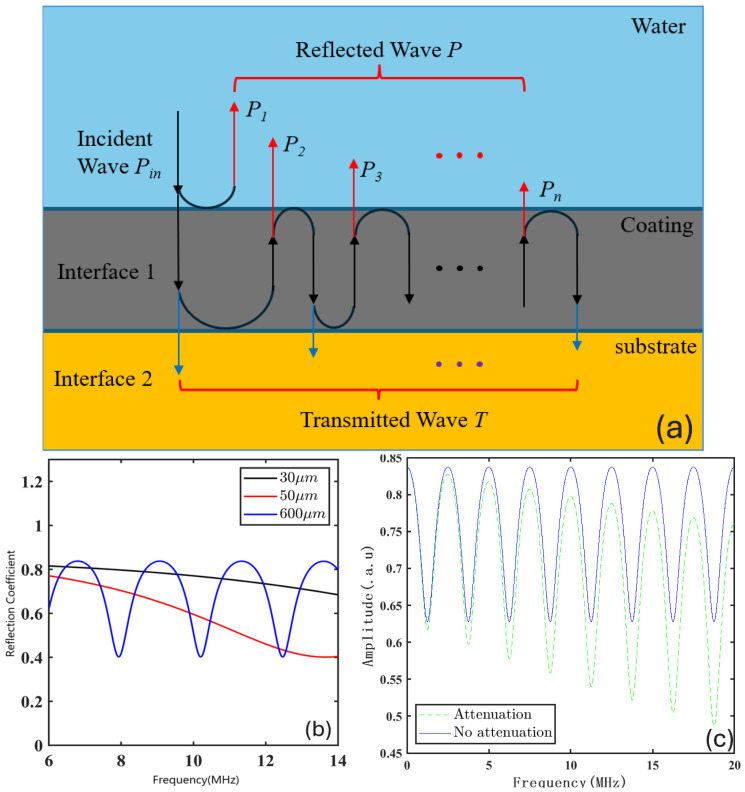
(**a**) Schematic diagram of ultrasonic wave reflection and transmission in coatings. (**b**) URCAS with and (**c**) without attenuation.

In this study, a water-immersion pulse–echo ultrasonic technique is employed to inspect the coating structure. The physical system can be simplified as a three-layer medium consisting of water, coating, and substrate, as shown in Figure 1a. Each layer is assumed to be homogeneous with parallel interfaces. When a plane ultrasonic wave $P_{1}$ is normally incident on the structure, multiple reflections and transmissions occur at the interfaces. The reflected signal in the water layer is the superposition of multiple propagation paths, corresponding to a round-trip reflection within the coating. Each additional round trip introduces extra phase accumulation and reflection/transmission coefficients [26].

(1) $$\begin{array}{ll} P_{1}= & r_{12}\exp\left(2ik_{1}z_{0}\right),\\ P_{2}= & r_{23}t_{12}t_{21}exp\left(2ik_{2}d+2ik_{1}z_{0}\right),\\ P_{3}= & r_{23}^{2}t_{12}r_{21}t_{21}\exp\left(4ik_{2}d+2ik_{1}z_{0}\right),\\ & \ldots \\ P_{n}= & r_{23}^{n}t_{12}r_{21}^{n-1}t_{21}\exp\left(2nik_{2}d+2ik_{1}z_{0}\right) \end{array}$$

where d denotes the coating thickness, while $k_{1}{,k}_{2}$ represent the wavenumbers of the ultrasonic wave in water and in the coating along the Z-axis direction, respectively. The coefficients $r_{12}=(\rho_{2}c_{2}-\rho_{1}c_{1})/(\rho_{2}c_{2}+\rho_{1}c_{1})$ and $r_{23}=(\rho_{3}c_{3}-\rho_{2}c_{2})/(\rho_{3}c_{3}+\rho_{2}c_{2})$ denote the acoustic pressure reflection coefficients at the water–coating interface and the coating–substrate interface, respectively. Similarly, $t_{12}$ and $t_{21}$ represent the acoustic pressure transmission coefficients for wave propagation from water into the coating and from the coating back into water, respectively. The summation of these contributions describes the superposition of all reflected waves, which forms the frequency-dependent interference pattern observed in the reflected signal, denoted as $P_{r}$. To avoid the phase interference introduced by the measurement system, the incident acoustic pressure $P_{in}$ is taken as the reference signal, and the acoustic pressure reflection coefficient of the coating is thereby obtained [27]:

(2) $$\;\;R\left(f\right)=\frac{P_{r}}{P_{in}}=\frac{r_{12}+r_{23}\exp\left(-2\alpha \left(f\right)d\right)\exp\left(i\varphi \left(f\right)\right)}{1+{r_{12}r}_{23}\exp\left(-2\alpha \left(f\right)d\right)\exp\left(i\varphi \left(f\right)\right)}$$

where f denotes the ultrasonic frequency, α(f) is the frequency-dependent attenuation coefficient of the coating, and φ(f) represents the phase shift induced by the coating. Since the acoustic pressure reflection coefficient of the coating is a complex quantity, it is not straightforward to analyze directly. Therefore, its magnitude is taken to extract amplitude information, yielding the ultrasonic reflection coefficient amplitude spectrum (URCAS), as shown in Figure 1b. The URCAS describes frequency-dependent interference caused by multiple reflections within the coating. It can be expressed as follows [27]:

(3) $$\begin{array}{c} \left|R\left(f\right)\right|={\left[\frac{r_{12}+r_{23}\exp{\left(-2\alpha \left(f\right)d\right)}^{2}-{4r_{12}r}_{23}\exp\left(-2\alpha \left(f\right)d\right){sin}^{2}\left(\frac{2\pi fd}{c_{2}}\right)}{1+{r_{12}r}_{23}\exp{\left(-2\alpha \left(f\right)d\right)}^{2}-{4r_{12}r}_{23}\exp\left(-2\alpha \left(f\right)d\right){sin}^{2}\left(\frac{2\pi fd}{c_{2}}\right)}\right]}^{\frac{1}{2}} \end{array}$$

For coatings with thickness much larger than the wavelength, the URCAS typically exhibits a series of periodic extrema (resonance peaks) induced by multiple-wave interference, which correspond to frequencies where the round-trip phase satisfies constructive or destructive interference conditions. These frequencies are given by $f_{n}=nc_{2}/4d$, where $f_{n}$ and n are the resonant frequency and the order of the resonant frequency, respectively, and $c_{2}$ denotes the acoustic velocity of the coating. By analyzing the positions of these resonance peaks, key parameters such as coating thickness or acoustic velocity can be conveniently determined. However, for the subwavelength coatings investigated in this study, defined as those with thickness smaller than one-tenth of the incident wavelength, the problem becomes significantly more challenging:

(1) The spectrum may contain only weak or no clear resonance extrema, as shown in Figure 1b, making conventional resonance-based methods ineffective;
(2) URCAS is highly sensitive to the attenuation coefficient α(f), which is usually unknown for industrial coatings. Inaccurate or neglected attenuation leads to distorted spectra and increased inversion error.

Therefore, although the URCAS provides valuable frequency-domain information, its strong dependence on attenuation and resonance features limits its reliability for subwavelength coatings.

### 2.2. The Layer-Phase Spectrum and Its Attenuation-Free Property

**Figure 2:**
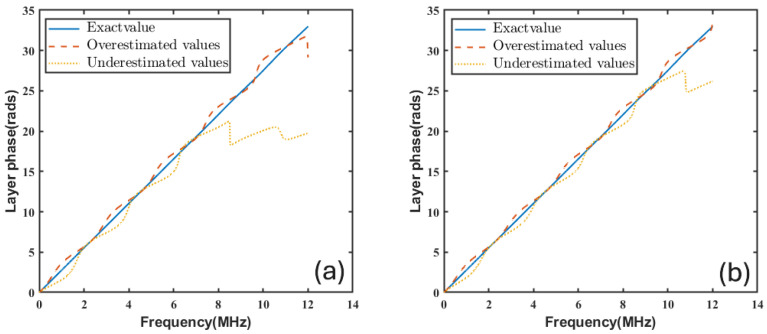
Nonlinear phenomena of layer phase spectrum: (**a**) curves for different acoustic velocities and (**b**) curves for different densities.

To reduce the dependence on attenuation, the layer-phase spectrum is introduced. The fundamental concept of the layer-phase spectrum is to extract the net phase delay arising solely from multiple reflections within the coating layer. When an ultrasonic wave propagates through the coating, a phase difference is generated between two consecutive reflected waves originating from the coating–substrate interface due to the round-trip propagation within the coating. This phase difference is defined as the layer phase. It is primarily governed by the propagation characteristics of the coating and is closely related to the physical properties of the coating, as shown in Figure 2. It can be expressed as [10]:

(4) $$\varphi_{L}\left(f\right)=\frac{4\pi df}{c_{2}}$$

The layer phase depends on thickness and acoustic velocity, but not on amplitude attenuation, making it less sensitive to unknown attenuation effects. This distinguishes it from amplitude-based methods, where attenuation directly affects spectral magnitude. However, in practical measurements, the phase directly extracted from the total reflected signal $P_{r}\;$ includes not only the phase contribution from the water–coating interface but also the inherent delay introduced by the measurement system. As a result, the layer phase $\varphi_{L}(f)$ cannot be directly obtained or utilized.

According to Equation (2), the layer-phase spectrum can be obtained from the phase difference between the total reflection coefficient and that of the water–coating interface [25]:

(5) $$\varphi_{L}\left(f\right)=\arg\left(\frac{R\left(f\right)-r_{12}}{1-r_{12}R\left(f\right)}\right)$$

This formulation removes attenuation-dependent terms and provides a more stable constraint for inversion, especially for subwavelength coatings.

### 2.3. Nonlinearity in the Layer Phase Spectrum

Section 2.2 shows that under ideal conditions (i.e., when the model parameters match the true physical properties), the layer-phase spectrum exhibits a linear relationship with frequency. In this section, we show that parameter mismatch introduces nonlinear distortion into the layer-phase spectrum and briefly explain the underlying mechanism. This provides a basis for using nonlinearity as an additional constraint in inversion. Equation (5) shows that the experimental extraction of the layer-phase spectrum, $\varphi_{L}(f)$, depends on the pressure reflection coefficient at the water/coating interface, $r_{12}$. During parameter inversion, this value is an estimate, $r_{12e}$, derived from the current guesses for acoustic velocity $c_{e}$ and density $\rho_{e}$. Substituting $r_{12e}$ with $r_{12}$, and combining Equations (2) and (5), the layer-phase spectrum can be expressed as:

(6) $$\;\;\varphi_{L}\left(f\right)=arg\frac{{(r}_{12a}-r_{12e})+(1-r_{12e}r_{12a})r_{23a}\exp\left(-2\alpha \left(f\right)d\right)\exp\left(ik_{e}f\right)}{(1-r_{12e}r_{12a})+{(r}_{12a}-r_{12e})r_{23a}\exp\left(-2\alpha \left(f\right)d\right)\exp\left(ik_{e}f\right)}$$

When the estimated acoustic impedance matches the true value (i.e., $r_{12a}=r_{12e}$), the layer-phase spectrum $\varphi_{L}(f)$ remains linear with respect to frequency. This deviation from linearity serves as a direct indicator of parameter inconsistency. When a mismatch occurs (i.e., $r_{12a}\neq r_{12e}$), the spectrum deviates from linearity. This behavior can be understood from Equations (7)–(9):

(7) $$\begin{array}{c} \left\{\begin{array}{c} N\left(f\right)={(r}_{12a}-r_{12e})+(1-r_{12e}r_{12a})r_{23a}exp(-2\alpha (f)d)exp(ik_{e}f)\\ D\left(f\right)=\left(1-r_{12e}r_{12a}\right)+(r_{12a}-r_{12e})r_{23a}exp(-2\alpha (f)d)exp(ik_{e}f) \end{array}\right. \end{array}\#$$

To evaluate the derivative of the above expression, we introduce the following notation:

(8) $$Z\left(f\right)=\frac{N\left(f\right)}{D\left(f\right)}$$

The derivative is then obtained by applying the chain rule, yielding:

(9) $$\begin{array}{c} Z^{\prime }\left(f\right)=\frac{D\left(f\right)\frac{dN\left(f\right)}{df}-N\left(f\right)\frac{dD\left(f\right)}{df}}{{\left|D\left(f\right)\right|}^{2}}\\ \frac{d\varphi \left(f\right)}{df}=\frac{Im\left(Z\prime \left(f\right)\bar{Z\left(f\right)}\right)}{{\left|Z\left(f\right)\right|}^{2}} \end{array}$$

These terms introduce coupling between frequency and inversion parameters, which breaks the ideal linear phase–frequency relationship. As a result, parameter mismatch leads to nonlinear distortion in the layer-phase spectrum. Therefore, the derivative $d\varphi \left(f\right)/df$ is no longer constant, and the layer-phase spectrum $\varphi \left(f\right)$ becomes nonlinear. This indicates that the nonlinearity of the layer-phase spectrum is mainly caused by impedance mismatch and can be used as an indicator of parameter inconsistency.

Conventional phase-based methods typically assume linear phase behavior and do not explicitly utilize this nonlinear characteristic, which limits their ability to distinguish parameter deviations.

To illustrate this effect, numerical simulations were conducted. Different sets of acoustic velocity and density (true, overestimated, and underestimated values) were used to compute the layer-phase spectrum. As shown in Figure 2, when the estimated parameters deviated from the true values, the layer-phase spectrum curves shifted from linear to nonlinear. Larger parameter deviations lead to more pronounced distortion.

These results confirm that the nonlinear behavior of the layer-phase spectrum provides additional information beyond linear phase variation, which can assist parameter inversion, especially for subwavelength coatings where amplitude features are limited.

### 2.4. Inversion Framework

The inversion of ultrasonic parameters is formulated as a nonlinear optimization problem, aiming to estimate thickness, acoustic velocity, and density by minimizing the discrepancy between measured and predicted spectra. The problem is characterized by strong nonlinearity and parameter coupling, especially due to the behavior of the layer-phase spectrum.

To systematically address this problem, a nonlinearity-guided inversion framework is proposed, consisting of: (i) the construction of a hybrid objective function incorporating both amplitude and phase information, (ii) the selection of an appropriate global optimization algorithm, and (iii) the development of a nonlinearity-guided update strategy to enhance inversion efficiency and robustness. The detailed formulation of each component is presented in the following subsections.

#### 2.4.1. Objective Function and Nonlinearity Quantification

Following the above framework, the inversion problem is first formulated through a hybrid objective function. The hybrid objective function combines the least-squares method and the correlation coefficient. Notably, the layer-phase-related term provides an additional constraint for inversion. The hybrid objective function is defined as follows:

(10) $$E_{\left|R\right|\varphi }\left(d,c_{2},\rho_{2}\right)=\frac{r_{A}+r_{B}}{\left(1+r_{P}\right)}$$

where $r_{A}$ represents the amplitude-spectrum constraint term, quantifying the least-squares difference between the measured and theoretical URCAS [28] as:

(11) $$r_{A}\left(d,c_{2},\rho_{2}\right)=\frac{\sum_{i=1}^{N}{\left(y_{i}\left(f,d,c_{2},\rho_{2}\right)-{y_{i}\left(f\right)}^{*}\right)}^{2}}{N}$$

where $y_{i}$ and $y_{i}^{*}$ are the theoretical and measured URCAS values at frequency point i, and N  is the total number of frequency points within the transducer’s effective bandwidth.

The term $r_{B}$ corresponds to the layer-phase spectrum constraint, which quantifies the least-squares difference for the layer-phase spectrum. Given its attenuation-independent nature, the layer-phase spectrum provides a more reliable and physically interpretable constraint, especially for subwavelength coatings where amplitude information is insufficient. Its difference is calculated as:

(12) $$\begin{array}{c} r_{B}\left(d,c_{2},\rho_{2}\right)=\frac{\sum_{i=1}^{N}{\left(\varphi_{Li}\left(f,d,c_{2}\right)-{\varphi_{Li}^{*}\left(f,c_{2},\rho_{2}\right)}^{*}\right)}^{2}}{N} \end{array}$$

where $\varphi_{Li}\left(f\right)=4\pi fd/c_{2}$ is the theoretical linear layer-phase spectrum, and $\varphi_{Li}^{*}$ is the layer-phase spectrum calculated from the measured data using the current estimates of $c_{2}$ and ρ.

The term $r_{P}$ denotes the correlation coefficient between the measured and theoretical URCAS. It is introduced to prevent optimization from focusing solely on amplitude magnitude errors while neglecting spectral shape consistency [29]:

(13) $$\;r_{p}\left(d,c_{2},\rho_{2}\right)=\frac{\sum_{i=1}^{N}\left[\left|y_{i}\left(f\right)\right|-\bar{\left|y_{\mathrm{i}}\left(f\right)\right|}\right]\left[\left|{y_{i}\left(f\right)}^{*}\right|-\bar{\left|{y_{\mathrm{i}}\left(f\right)}^{*}\right|}\right]}{\sqrt{\sum_{i=1}^{N}{\left[\left|y_{i}\left(f\right)\right|-\bar{\left|y_{\mathrm{i}}\left(f\right)\right|}\right]}^{2}\sum_{i=1}^{N}{\left[\left|y_{i}\left(f\right)\right|-\bar{\left|y_{\mathrm{i}}\left(f\right)\right|}\right]}^{2}]}}$$

To explicitly quantify the nonlinearity of the layer-phase spectrum, the sum of squared residuals (SSR) obtained from a linear fit to the phase spectrum is introduced. Specifically, the layer-phase spectrum is fitted using a linear model with respect to frequency, and the SSR is defined as:

(14) $$SSR=\sum_{i=1}^{n}{e_{i}}^{2}=\sum_{i=1}^{n}({\varphi_{Li}-\hat{\varphi_{Li}})}^{2}$$

where $\hat{\varphi_{Li}}$ denotes the fitted linear phase, and $\varphi_{Li}$ represents the calculated layer-phase spectrum. The SSR thus quantifies the deviation from ideal linear behavior, with larger values indicating stronger nonlinearity.

Importantly, although the layer-phase term is formulated as a least-squares difference, it implicitly introduces a nonlinearity constraint. Therefore, minimizing this term is equivalent to suppressing the nonlinearity of the phase spectrum, thereby enforcing physical consistency of the inversion parameters. It should be noted that the proposed method does not remove the physical effect of attenuation but mitigates its impact on inversion by incorporating attenuation-insensitive spectral features.

#### 2.4.2. Selection of Optimization Algorithm

Based on the characteristics of the objective function defined above, an appropriate optimization algorithm is required. The proposed inversion problem is a strongly nonlinear and highly coupled parameter estimation task based on the joint inversion of amplitude spectra and layer-phase spectra. The objective function in this study involves multiple unknowns, including coating thickness, sound speed, and density. In addition, due to the nonlinear dependence of the phase spectrum on material parameters, the objective function landscape may contain multiple local optima. Therefore, a derivative-free global optimization strategy is required.

To determine the appropriate optimizer, several algorithms were considered, including Red Fox Optimization (RFO), Differential Evolution (DE), Grey Wolf Optimizer (GWO), and Sailfish Optimization Algorithm (SFOA), as well as Particle Swarm Optimization (PSO) [30]. These algorithms were evaluated under the same settings, including the same population size, number of iterations, and parameter ranges, to ensure a fair comparison. The convergence curves show that PSO achieves faster convergence to lower objective values and exhibits more stable convergence behavior compared with the other algorithms.

**Figure 3:**
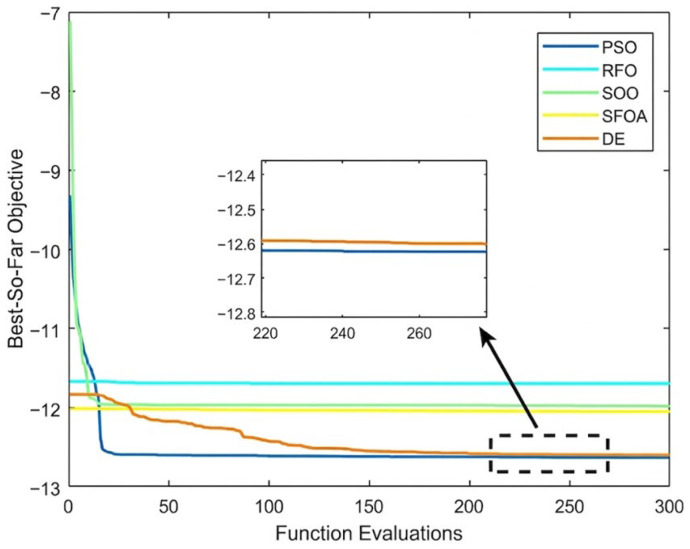
Comparison results of different optimization algorithms.

The Rastrigin function is used as a general benchmark to evaluate optimization behavior, rather than to represent the specific inversion problem. To ensure a fair comparison among different optimization algorithms, the same search space, population size, function-evaluation budget, and number of independent runs were used in all tests. The corresponding convergence curves are presented in Figure 3, demonstrating the comparative performance of the considered algorithms. These results indicate that PSO is well suited to exploit the nonlinear sensitivity of the dual-spectrum features while maintaining robust global search capability.

#### 2.4.3. Nonlinearity-Guided PSO Algorithm

Based on the selected optimization algorithm, a nonlinearity-guided PSO strategy is further developed to fully exploit the physical meaning of the layer-phase nonlinearity. In standard PSO, each particle i  has a position $x_{i}\;$ (representing a potential solution $\left[d,c_{2},\rho \right]$) and a particle velocity $v_{i}$. The particle’s movement is guided by its personal best position ($p_{{\mathrm{best}}_{i}}$) and the swarm’s global best position ($g_{\mathrm{best}}$):

(15) $$v_{id}^{t+1}=w\;v_{id}^{t}+c_{1}\;r_{1}\left(p_{besti}-x_{id}\right)+c_{2}\;r_{2}\left(G_{besti}-x_{id}\right)$$

(16) $$x_{id}^{t+1}=x_{id}^{t}+v_{id}^{t+1}$$

To enhance exploration and prevent premature convergence, we introduce a random mutation factor μ·δ to the velocity update and a perception factor η·γ to the position update:

(17) $$v_{id}^{t+1}=w\;v_{id}^{t}+c_{1}\;r_{1}\left(p_{besti}-x_{id}\right)+c_{2}\;r_{2}\left(G_{besti}-x_{id}\right)+\mu \cdot \delta$$

(18) $$x_{id}^{t+1}=x_{id}^{t}+v_{id}^{t+1}+\eta \cdot \gamma$$

The key idea is to use the nonlinearity of the layer-phase spectrum to guide parameter updates. The nonlinearity in the calculated layer-phase spectrum is primarily caused by inaccuracies in the estimated acoustic impedance $Z_{c}=\rho c_{2}$, which depends on density and acoustic velocity but is independent of thickness. The degree of nonlinearity, quantified by the SSR defined in Section 2.4.1, is used here as a key indicator to guide the optimization process.

Case 1 (Strong nonlinearity): If the current SSR is higher than both the personal and global best SSRs, it indicates a significant error in $Z_{c}$. The particle updates all three parameters ($d,c_{2},\rho$) towards the personal and global bests.

(19) $$\begin{array}{ll} v_{id}^{t+1} & =w\;v_{id}^{t}+c_{1}\;r_{1}\left(p_{besti}\left(d,c_{2},\rho_{2}\right)-x_{id}\left(d,c_{2},\rho_{2}\right)\right)\\ & +\;c_{2}\;r_{2}\left(G_{besti}\left(d,c_{2},\rho_{2}\right)-x_{id}\left(d,c_{2},\rho_{2}\right)\right) \end{array}$$

Cases 2 and 3 (Moderate and slight nonlinearity): If the current SSR is better than one but worse than the other, it updates a combination of the thickness and the $Z_{c}$-related parameters ($c_{2},\rho$).

(20) $$\begin{array}{ll} v_{id}^{t+1} & =w\;v_{id}^{t}+c_{1}\;r_{1}\left(p_{besti}\left(d,c_{2},\rho_{2}\right)-x_{id}\left(d,c_{2},\rho_{2}\right)\right)\\ & +\;c_{2}\;r_{2}\left(G_{besti}\left(d\right)-x_{id}\left(d\right)\right) \end{array}$$

or

(21) $$\begin{array}{ll} v_{id}^{t+1} & =w\;v_{id}^{t}+c_{1}\;r_{1}\left(p_{besti}\left(d\right)-x_{id}\left(d\right)\right)\\ & +\;c_{2}\;r_{2}\left(G_{besti}\left(d,c_{2},\rho_{2}\right)-x_{id}\left(d,c_{2},\rho_{2}\right)\right) \end{array}$$

Case 4 (Weak nonlinearity): If the current SSR is lower than both, it suggests the $Z_{c}$ is accurate, and that the main error lies in thickness. The particle then updates only the thickness dimension (d), while $c_{2}$ and ρ remain unchanged.

(22) $$\begin{array}{ll} v_{id}^{t+1} & =w\;v_{id}^{t}+c_{1}\;r_{1}\left(p_{besti}\left(d\right)-x_{id}\left(d\right)\right)\\ & +\;c_{2}\;r_{2}\left(G_{besti}\left(d\right)-x_{id}\left(d\right)\right) \end{array}$$

**Figure 4:**
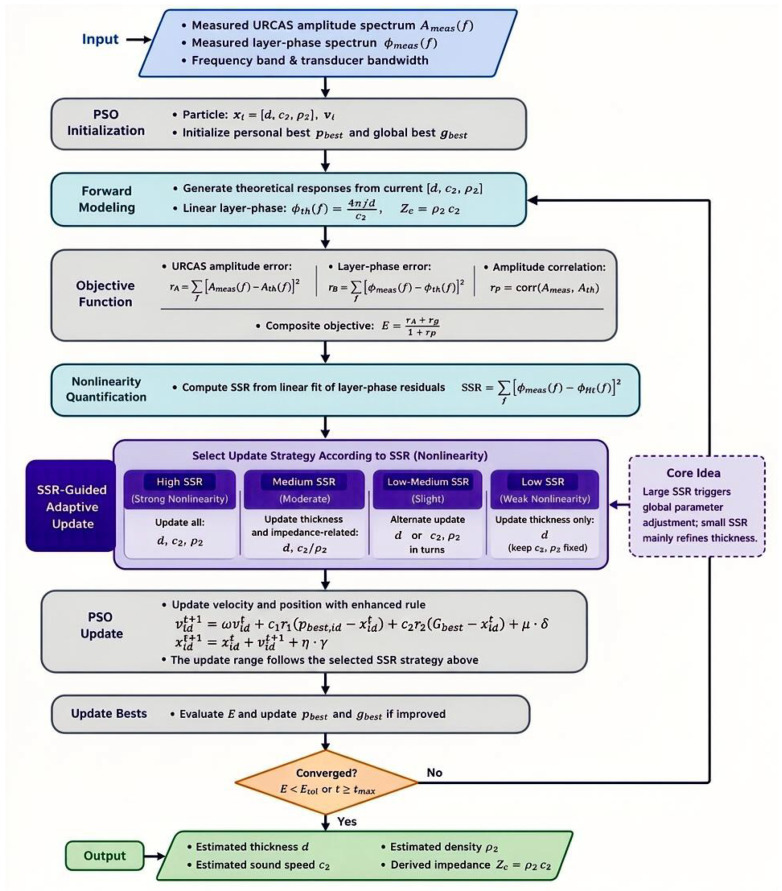
Iteration flowchart for parameter inversion.

This strategy enables adaptive parameter updating by linking the optimization behavior to the physical meaning of nonlinearity: large global adjustments are performed when the impedance mismatch is significant (strong nonlinearity), while fine-tuning is focused on thickness when the impedance is close to the true value (weak nonlinearity). The complete algorithm workflow is illustrated in Figure 4.

## 3. Finite-Element Simulation and Analysis

A series of finite-element simulations were conducted to verify the feasibility and effectiveness of the proposed method.

### 3.1. Model Setup and Material Parameters

Finite-element (FE) modeling of ultrasonic wave propagation was performed using COMSOL6.2 Multiphysics. A two-dimensional (2D) cross-sectional model was established to represent the water–coating–substrate system under normal-incidence excitation. The geometry of the simulation model is illustrated in Figure 4, with a model width of 2.0 mm. The 2D approximation was adopted because this study focuses on longitudinal wave propagation, multiple reflections, and spectral interference along the thickness direction. Under the plane-wave assumption, the main features of URCAS and the layer-phase spectrum can be captured within this framework. In addition, the lateral dimensions of the transducer and coating are much larger than the ultrasonic wavelength, so out-of-plane variations are relatively weak. However, the 2D model cannot capture a full three-dimensional effect, such as beam spreading, edge diffraction, and lateral energy distribution. To address these limitations, additional simulations with non-planar wave fields will be further discussed.

**Figure 5:**
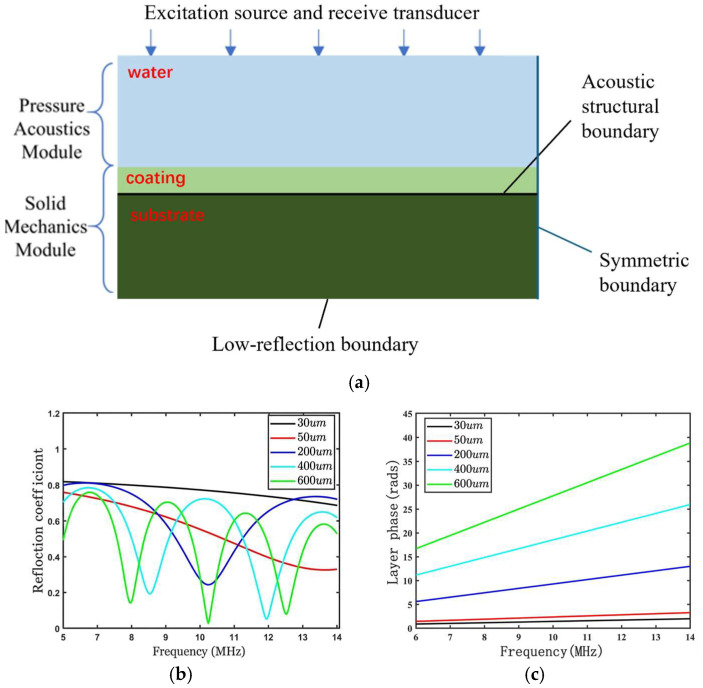
FE simulation: (**a**) model setup, (**b**) URCAS, and (**c**) layer-phase spectrum for different coating thicknesses.

The coating material is PTFE, and the substrate is aluminum alloy. The material properties used in the simulations are listed in Table 1. The center frequency of incident wave is 10 MHz, and the corresponding wavelength in PTFE is 273.2 μm. For comparative analysis, the coating thickness was set as 600 μm (approximately 2.19 wavelengths), 400 μm (1.46 wavelengths), 200 μm (0.73 wavelengths), 50 μm (0.18 wavelengths), and 30 μm (0.11 wavelengths), covering both multi-wavelength and subwavelength regimes. The attenuation coefficient within PTFE is set as 0.421 Np/mm. The detailed geometry and boundary conditions are shown in Figure 5a.

In the FE model, the coupling medium (water) was described using the pressure acoustics module, while the coating and aluminum substrate were modeled using the solid mechanics module. The water–coating interface was defined as an acoustic–structure coupling boundary. Symmetry boundary conditions were applied on the left and right sides of the model to suppress artificial reflections. The bottom of the aluminum substrate was assigned a low-reflection boundary, while the top boundary was set as excitation and reception. The excitation signal was defined as a cosine-modulated pulse with a center frequency of 10 MHz and a −6 dB bandwidth ranging from 6.68 to 13.3 MHz. To ensure numerical accuracy, the mesh size and time step were selected according to the Courant–Friedrichs–Lewy (CFL) condition [31]. Specifically, the maximum mesh size in the coating region was set to 1 μm, while a mesh size of 5 μm was used elsewhere. The time step was set to 1 ns.

### 3.2. Received Signals and Their Spectra

For thicknesses of 400 μm and 600 μm, the time-domain signals clearly separate the surface and bottom echoes. When the coating thickness is smaller than 200 μm, the echoes significantly overlap, making it difficult to distinguish surface and interface reflections in the time domain. The signals were transformed into the frequency domain using the Fourier transform. The URCAS and layer-phase spectrum are presented in Figure 5b and Figure 5c, respectively. From the URCAS, the 600 μm-thick coating exhibits multiple distinct resonance features at frequencies of 7.95 MHz, 10.22 MHz, and 12.49 MHz. As the thickness decreases, the number of observable resonance features is reduced. The 50 μm-thick coating shows only one resonance feature, while no clear resonance is observed for the 30 μm coating. This indicates that thinner coatings provide less spectral information, which makes inversion more challenging. The layer-phase spectrum exhibits an approximately linear dependence on frequency. Its slope can be used to estimate thickness or acoustic velocity.

### 3.3. Comparative Analysis with URCAS

With the attenuation coefficient set to zero, parameter inversion was performed using both the conventional URCAS method and the proposed nonlinearity-guided approach. For multi-parameter inversion, the mean relative error (MRE) is used to evaluate overall accuracy, defined as:

(23) $$MRE=\frac{1}{N}\sum_{i=1}^{N}\frac{x_{i}^{ture}-x_{i}^{inv}}{x_{i}^{ture}}$$

where *N* denotes the number of parameters, and $x_{i}^{\mathrm{true}}$ and $x_{i}^{\mathrm{inv}}$ represent the true and the estimated values of the i-th parameter, respectively.

To verify the validity of each component of the objective function described in Equation (10), several alternative objective functions were constructed and compared. Their performance was evaluated using the MRE obtained from simulations with coating thicknesses of 30 μm, 50 μm, and 600 μm, covering both subwavelength and super-wavelength regimes. The results in Table 2 show that the objective functions based on individual components exhibit higher MRE values than the combined formulation. This indicates that each component contributes to the overall inversion performance. The combined objective function achieves the lowest MRE.

In addition to inversion accuracy, the computational efficiency of the proposed nonlinearity-guided PSO algorithm was also evaluated. To provide a fair comparison, the 600 μm simulation model was repeatedly inverted 20 times using both the proposed and standard PSO under identical settings. The average number of iterations and total running time are summarized in Table 3.

The proposed method requires fewer iterations on average, indicating faster convergence. Although the proposed strategy introduces additional calculations in each iteration, the reduced parameter search range leads to a lower overall computational cost. Consequently, the total running time is reduced by 1.48 s compared with the standard PSO. These results suggest that the proposed strategy can improve computational efficiency under the tested conditions.

**Figure 6:**
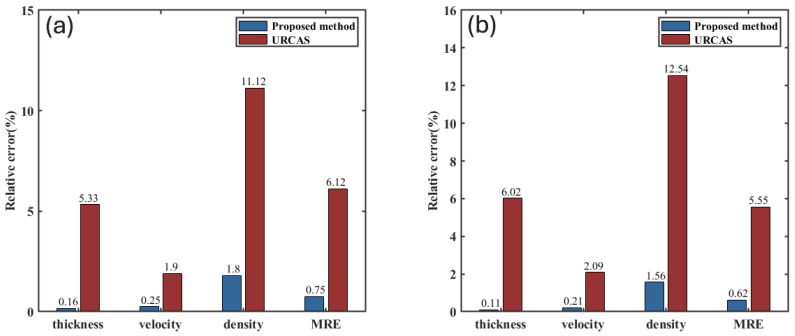

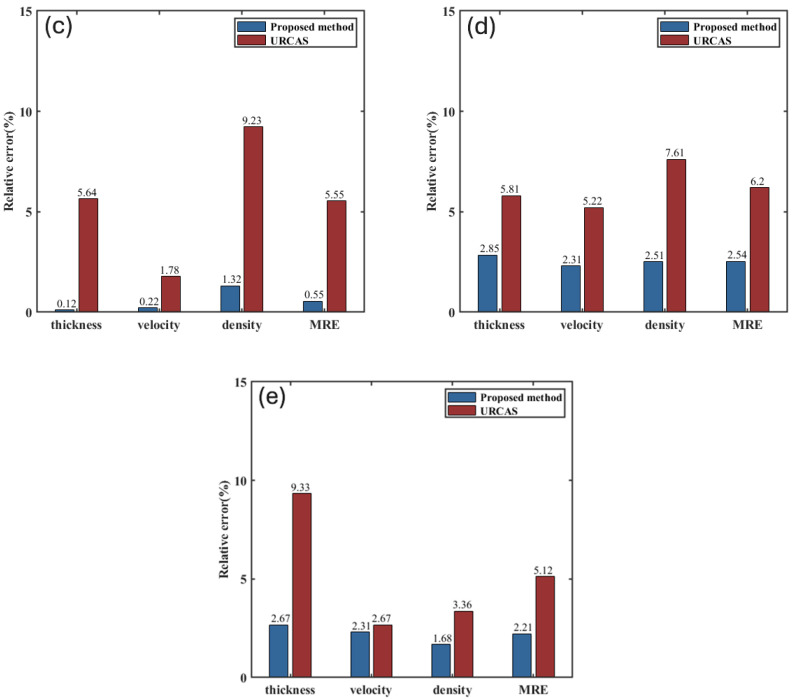
Comparison of absolute relative error in simulation for thicknesses of (**a**) 600 μm, (**b**) 400 μm, (**c**) 200 μm, (**d**) 50 μm, and (**e**) 30 μm.

Based on the designed objective function, a comparison of inversion errors between the proposed method and the conventional URCAS method for coatings with different thicknesses is presented in Figure 6. The results show that the MRE of the traditional URCAS method exceeds 5% for all five cases.

In comparison, the proposed method yields lower errors, with all parameters below 3% in the tested cases. For relatively thick coatings (e.g., 600 μm, 400 μm, and 200 μm), both individual parameter errors and MRE are below 2%. When resonance features are present, the proposed method appears less sensitive to unknown attenuation, allowing attenuation to be neglected in these cases. For the 30 μm coating, the error increases slightly due to the absence of clear resonance features but remains within 3%. Overall, the proposed method provides more stable inversion results than the conventional URCAS approach under the considered conditions without requiring prior knowledge of attenuation.

### 3.4. Influence of Non-Planar Wave Field and Water Distance

Although the analysis in Section 2.1, Section 2.2, Section 2.3, Section 2.4, Section 3.1, Section 3.2 and Section 3.3 is based on a plane-wave assumption for simplicity, practical ultrasonic measurements involve finite-aperture transducers that generate non-planar fields. To evaluate the sensitivity of the proposed method under realistic conditions, the plane wave assumption is relaxed, and a non-planar wave field is considered. In practice, the acoustic field depends on the transducer aperture and the water standoff distance, leading to spatial variations in amplitude and phase.

**Figure 7:**
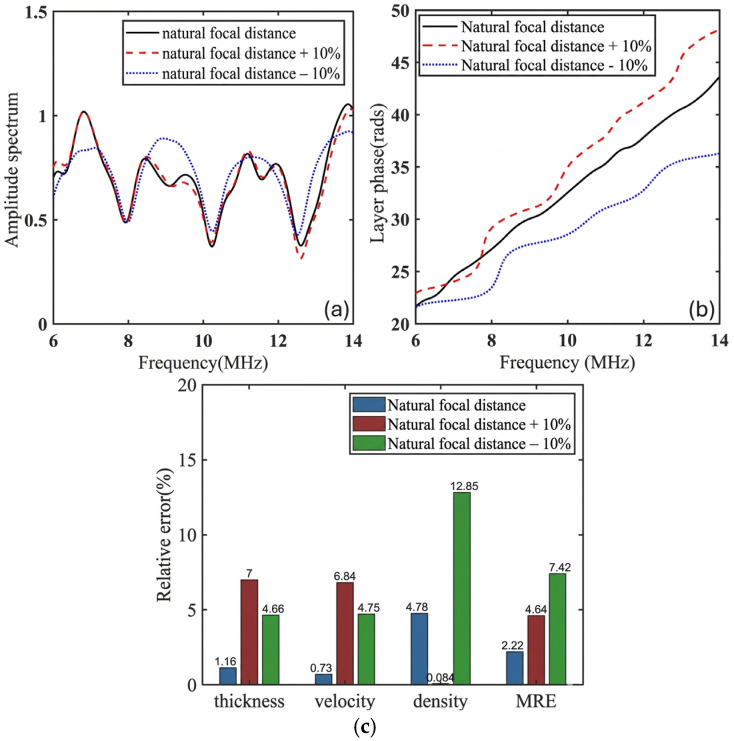
Results under different water distances. (**a**) Amplitude spectra (**b**) layer phase spectra of water distance testing model, and (**c**) the inversion results.

In this study, a non-planar wave propagation model is introduced to investigate the influence of water distance deviation from the natural focal distance. The simulation results are shown in Figure 7. When the water distance changes, both the arrival time and amplitude vary. At the natural focal position, the surface echo reaches its maximum amplitude, while the substrate echo is relatively weaker due to energy redistribution.

Compared with the plane-wave case, the non-planar wave field introduces additional effects such as diffraction, wavefront curvature, and weak mode conversion. These effects slightly perturb the amplitude spectrum and layer-phase spectrum, resulting in small local variations. The corresponding inversion deviations are shown in Figure 7c.

At the focal position, the inversion results remain stable, with relative errors below 5%. This indicates that the proposed method can tolerate realistic beam profiles to some extent. When the water distance deviates from the natural focal position by ±10%, the inversion error increases. In particular, the case of −10% deviation exhibits larger errors than the +10% case, indicating asymmetric sensitivity to defocusing. This behavior is likely related to reduced signal coherence under negative defocusing conditions.

Overall, compared with the ideal plane-wave model, the non-planar wave field introduces a moderate increase in inversion error; however, the accuracy remains within an acceptable range. These results demonstrate that the proposed method does not rely strictly on the plane-wave assumption. For experimental implementation, adjusting the water distance close to the focal position is sufficient to achieve stable inversion results.

### 3.5. Noise Robustness Evaluation

In practical ultrasonic signal acquisition, noise is an unavoidable factor that degrades signal quality, leading to deviations between measured and ideal signals. To investigate the influence, Gaussian white noise was added to the ultrasonic signals in the simulations. The Gaussian white noise is defined as follows:

(24) $$P\left(x\right)=\frac{1}{\sqrt{2\pi \sigma^{2}}}e^{-\frac{x^{2}}{2\sigma^{2}}}$$

where $\sigma^{2}=\frac{sigPower}{SNR}$ denotes the variance of the noise; sigPower represents the average power of the signal; and SNR denotes the signal-to-noise ratio. Noise levels of 10 dB, 20 dB, and 30 dB were considered.

**Figure 8:**
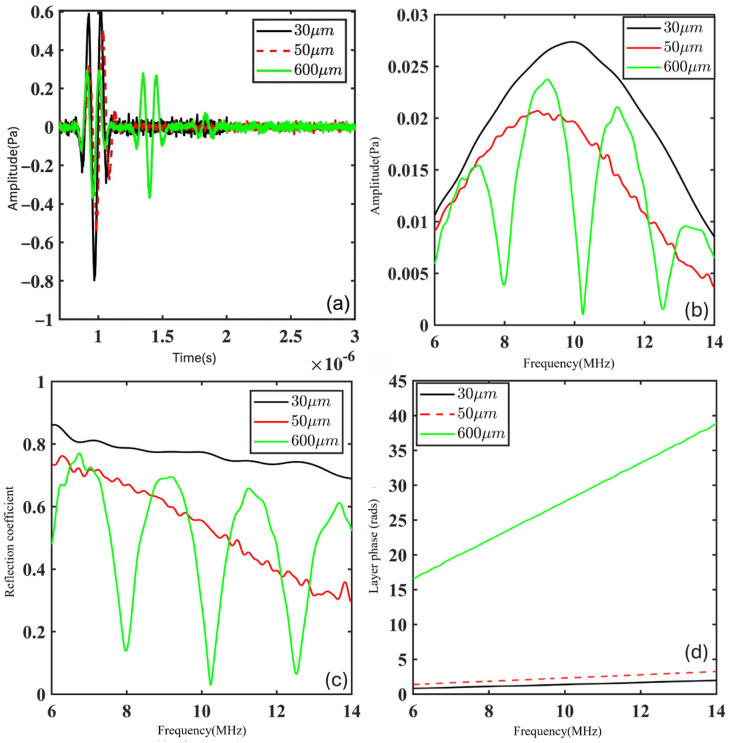
Influence of noise analysis: (**a**) waveform, (**b**) frequency spectra, (**c**) URCAS, and (**d**) layer phase spectrum with 20 dB Gaussian white noise.

Noise introduces distortions in the spectra, affecting both the URCAS and the layer-phase spectrum. As illustrated in Figure 8c, the URCAS exhibits noticeable fluctuations under noise. The layer phase spectrum is also affected, as shown in Figure 8d; however, the fluctuations are relatively smaller.

**Figure 9:**
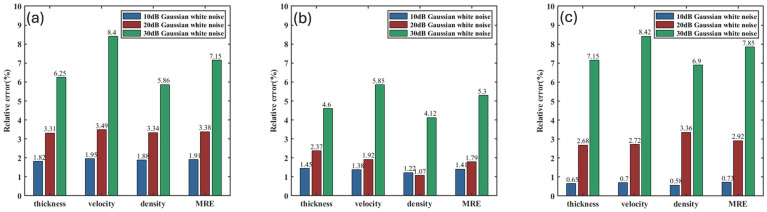
Inversion results for the thickness of (**a**) 600 μm, (**b**) 50 μm, and (**c**) 30 μm with 10, 20, and 30 dB noises.

The inversion results are presented in Figure 9. The effect of noise on inversion accuracy is not strictly monotonic, especially for very thin coatings. For the 600 μm model, the mean relative error (MRE) increases from 1.91% to 3.38% and 7.15% as the SNR changes from 10 dB to 20 dB and 30 dB, indicating performance degradation with increasing noise. A similar trend is observed for the 50 μm coating, where the MRE increases from 1.4% to 1.79% and 5.3%.

For the 30 μm coating, the MRE does not follow a simple trend. The values are 0.73%, 2.92%, and 7.85% at 10 dB, 20 dB, and 30 dB, respectively. This may be related to strong parameter coupling and weak spectral features at very small thicknesses. In such cases, a certain level of noise may help the optimization avoid getting trapped in local solutions.

Overall, the proposed method keeps the MRE below 8% for all tested SNR levels (10–30 dB) and thicknesses (30–600 μm). The error increases with noise, but the change is moderate. This suggests that the proposed method maintains reasonable performance under noisy conditions. The improved stability is likely related to the use of the layer-phase spectrum, which is less sensitive to amplitude fluctuations.

## 4. Experimental Validations

### 4.1. Coating Samples and Reference Measurements

**Figure 10:**
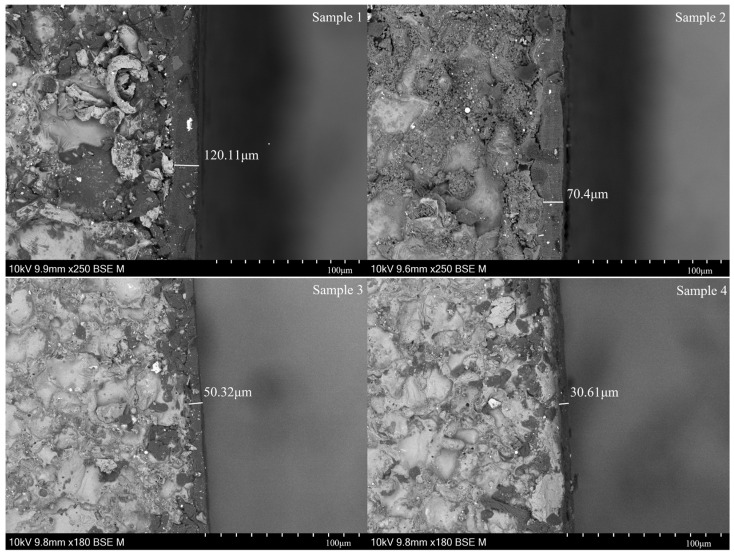
Cross-sectional images of four coating samples.

The experimental samples were single-layer coated aluminum alloy plates provided by the manufacturer. A total of eight samples were prepared on aluminum substrates with a thickness of 2 mm. The coatings were prepared using a thermal spraying process. The coating thickness was measured using a Scanning Electron Microscope (SEM) as the reference method, while the density was determined using the Archimedes method. The cross-sectional morphology of four representative coatings is shown in Figure 10.

The acoustic velocity of the coating was measured using an ultrasonic time-of-flight (TOF) method based on the Hilbert transform, applied to the thickest sample. An ultrasonic transducer with model HM1002-8 was employed with a center frequency of 68.57 MHz and a relative bandwidth of 37.2%.

The measured coating thicknesses for Samples 1–8, are 120.11 μm, 70.40 μm, 50.32 μm, 30.61 μm, 58.05 μm, 36.83 μm, 42.90 μm, and 20.89 μm. Sample 1 corresponds to a thickness-to-wavelength ratio of 0.55, while Sample 8 is the thinnest (0.095), for which weak resonance features are expected. Therefore, the samples cover a typical subwavelength thickness range. The acoustic velocity and density of the coating material were measured as 2303.23 m/s and 1682.76 kg/m^3^, respectively. For the aluminum thickness substrate, the thickness, acoustic velocity, and density were 1990.33 μm, 6032 m/s, and 2700 kg/m^3^, respectively. These samples provide a representative experimental range for evaluating the proposed method.

### 4.2. Ultrasonic C-Scan System and Data Collection

**Figure 11:**
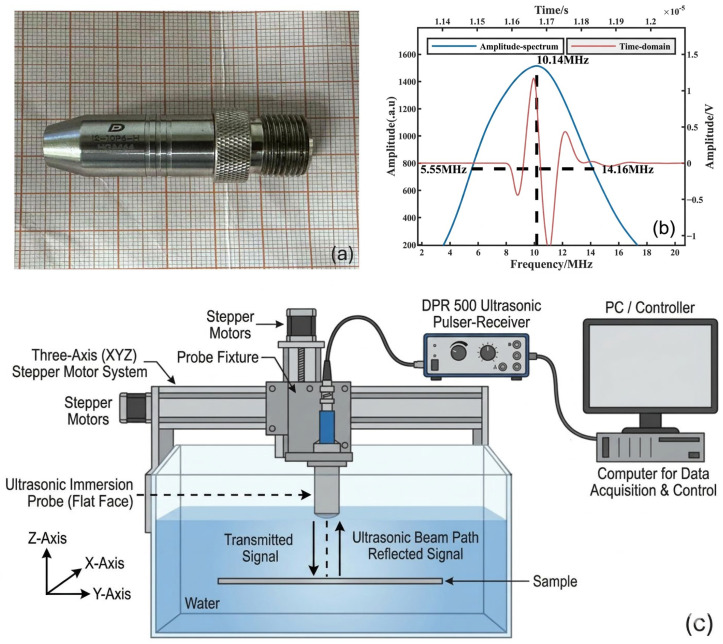
(**a**) Ultrasonic transducer with (**b**) its waveform and spectrum and (**c**) experiment system.

The 10 MHz ultrasonic transducer (I2-10P6-H, Doppler, Guangzhou, China) was used for data collection, as shown in Figure 11a,b. The time-domain waveform was transformed into the frequency domain, yielding a center frequency of 10.14 MHz and a −6 dB bandwidth ranging from 5.55–14.16 MHz. The experiments were conducted using a water-immersion ultrasonic inspection system, as shown in Figure 11c. The system mainly consists of a three-axis motion platform with a positioning accuracy of 5 μm, a JSR DPR500 pulser/receiver (Imaginant Inc, NY, USA), and a DSO054A oscilloscope (Keysight, CA, USA).

The inspection area was controlled within the near-field region of the ultrasonic transducer. In the near-field region, the ultrasonic wave can be approximated as quasi-planar, allowing for the application of ideal plane wave models. The scan area was set to 10 mm × 10 mm with a step size of 0.2 mm.

**Figure 12:**
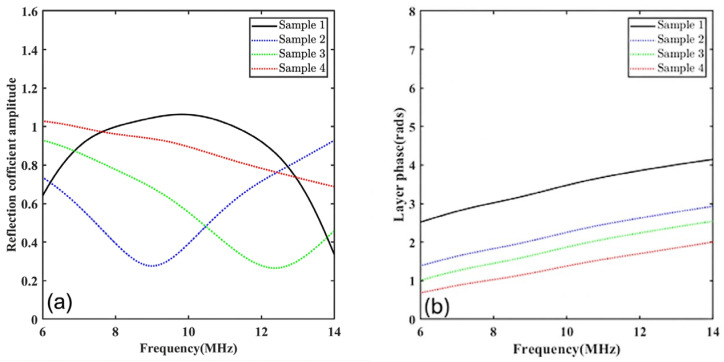
Measured signal for four coating samples: (**a**) URCAS and (**b**) layer-phase spectrum.

To improve the SNR, each signal was averaged over 256 acquisitions. The reflection from the water/aluminum interface was measured as the reference signal to calculate URCAS. Maintaining a constant transducer-to-surface distance, the reflection signals were subsequently acquired. Figure 12a,b presents the typical URCAS and layer-phase spectra of Samples 1–4. Consistent with the simulation results, thicker coatings exhibit more distinguishable spectral features, while thinner coatings show weaker or less pronounced characteristics. The corresponding C-scan inversion results are presented in the following section to evaluate the method’s performance.

### 4.3. Inversion Results and Error Analysis

The multiparameter inversion of the coating was performed for scanned areas of all eight specimens using both the conventional URCAS method and the proposed method. For clarity, C-scan results are shown for a representative sample (Sample 1), while statistical results for all samples are summarized in Table 4.

**Figure 13:**
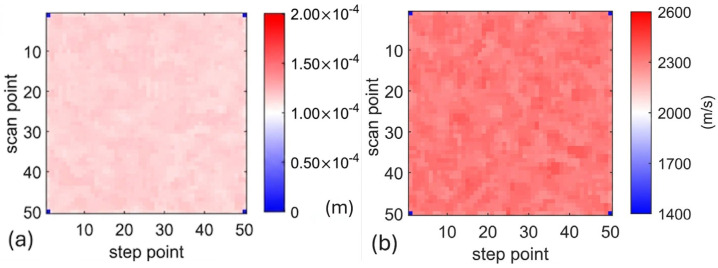

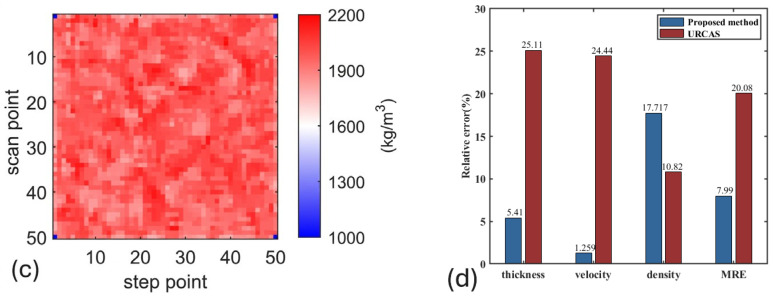
C-scan imaging of (**a**) thickness, (**b**) acoustic velocity, (**c**) density, and (**d**) relative error comparison between the proposed method and URCAS method for Sample 1.

Figure 13 shows the reconstructed results of thickness, acoustic velocity, and density for Sample 1. The thickness image indicates relatively small spatial variations, demonstrating a relatively uniform thickness. In contrast, larger fluctuations are observed in acoustic velocity and density, which may be attributed to material inhomogeneity.

The relative errors between the averaged inversion results and the reference values are shown in Figure 13d. Compared with the URCAS method, the proposed method yields lower errors in thickness and acoustic velocity, while the density error remains relatively higher. The resulting MRE is below 8%.

The statistical inversion results for all eight samples are summarized in Table 4. The MRE ranges from 3.17% to 9.85%, covering a thickness range of 20–114 μm. Compared with the URCAS-only method, a general improvement in accuracy is observed.

A thickness-dependent trend can be identified. For relatively thicker coatings (Samples 1–3), the MRE remains below 8%. For intermediate thicknesses (Samples 4–6), the MRE increases slightly. This is because the spectral features become weaker, and parameter coupling becomes stronger. For the thinnest samples (Samples 7–8), higher errors are observed, reaching up to 9.85%. The standard deviations of acoustic velocity and density are larger than those of thickness, indicating higher sensitivity to noise and parameter coupling. Overall, the MRE is below 10% for all samples. The results show that the proposed method maintains reasonable accuracy across the tested subwavelength range, although performance degrades as the thickness decreases.

Several limitations of the proposed method should be noted. The effectiveness of the method depends on the reflection coefficient at the interface. For low acoustic impedance contrast, both amplitude and phase spectral features become less pronounced, which may reduce the SNR and increase inversion instability. Moreover, when the coating thickness becomes extremely small relative to the wavelength, the inversion problem may become mathematically ill-posed due to insufficient independent spectral information. Multiple reflections and possible dispersion effects may also affect the extracted spectra. At last, residual errors may also arise from experimental noise, slight deviations from the plane-wave assumption, and material inhomogeneity introduced during the coating fabrication process. These effects become more pronounced for ultra-thin coatings.

## 5. Conclusions

This paper investigated the simultaneous nondestructive characterization of thickness, density, and acoustic velocity for subwavelength coatings through a combination of numerical simulations and experimental validations. The proposed method integrates the ultrasonic reflection coefficient amplitude spectrum (URCAS) with the layer-phase spectrum, guided by the observation that the layer-phase spectrum exhibits distinct nonlinearity when the assumed acoustic velocity and density deviate from their true values. This nonlinearity is exploited to develop a nonlinearity-guided particle swarm optimization (PSO) algorithm that dynamically adjusts its parameter update strategy, enabling robust multiparameter inversion without any prior knowledge of acoustic attenuation. The main conclusions are as follows:

(1) For subwavelength coatings with overlapping time-domain signals, the combined use of URCAS and the layer-phase spectrum enables simultaneous inversion of thickness, acoustic velocity, and density. This method reduces the dependence on acoustic attenuation, which is difficult to measure for thin coatings, and improves parameter identifiability under weak spectral features.
(2) Simulations demonstrate that the proposed method achieves lower inversion errors compared with the conventional URCAS-only approach. The accuracy decreases as the coating thickness is reduced, reflecting the reduced spectral sensitivity in the subwavelength regime.
(3) Experimental validation on PTFE coatings demonstrates that the method maintains mean relative errors below 10% across the tested samples. Larger deviations are observed for density, likely due to material inhomogeneity and experimental uncertainties.

Despite these promising results, several limitations remain. The performance of the proposed method depends on sufficient spectral contrast, and its performance may degrade for extremely thin coatings or materials with low acoustic impedance contrast. In addition, deviations from ideal assumptions and experimental noise may introduce additional uncertainties in the inversion results. Future work will focus on extending the inversion framework to more complex coating conditions and further validating its applicability across a broader range of materials and experimental scenarios.

## Figures and Tables

**Table 1:** The material properties used in the FE simulations.

Material	Thickness (μm)	Velocity (m/s)	Density (kg/m^3^)
Water	600	1481	1000
PTFE	600/400/200/50/30	2732	1190
Aluminum	100	6032	2700

**Table 2:** MRE comparison of different objective functions.

Object Function	30 μm	50 μm	600 μm
$r_{A}$	3.12	5.34	15.31
$\frac{1}{1+r_{p}}$	20.21	5.88	3.94
$\frac{r_{A}}{1+r_{p}}$	12.40	15.50	10.50
$\frac{r_{B}}{1+r_{p}}$	4.00	4.44	35.30
$\frac{r_{A}r_{B}}{1+r_{p}}$	5.72	5.88	34.41
$\frac{r_{A}+r_{B}}{1+r_{p}}$	2.92	1.79	3.38

**Table 3:** Performance comparison between standard and proposed PSO.

Algorithm	Average Iterations	Average Runtime (s)
Standard PSO	383	10.52
Proposed PSO	321	8.94

**Table 4:** Statistical inversion results of the eight coating samples.

Samples	Thickness (μm)	Acoustic Velocity (m/s)	Density (kg/m^3^)	MRE
1	114.16 ± 9.11	2274.53 ± 217.31	1980.61 ± 301.41	7.99
2	68.67 ± 10.65	2496.75 ± 115.33	1624.14 ± 81.68	3.17
3	47.41 ± 9.68	2367.15 ± 479.41	1680.15 ± 297.14	3.6
4	29.14 ± 6.24	2150.01 ± 456.47	1470.59 ± 528.86	6.53
5	54.91 ± 5.48	1720.43 ± 176.14	1691.76 ± 197.24	8.41
6	40.32 ± 5.74	2119.32 ± 229.00	1918.29 ± 258.33	5.98
7	49.20 ± 7.68	1783.86 ± 218.12	1730.64 ± 298.14	7.46
8	20.72 ± 7.68	1690.21 ± 185.05	1680.14 ± 199.91	9.85

## Data Availability

Data will be made available only on reasonable request.
